# The Barriers to Deprescription in Older Patients: A Survey of Spanish Clinicians

**DOI:** 10.3390/healthcare11131879

**Published:** 2023-06-29

**Authors:** Marta Mejías-Trueba, Aitana Rodríguez-Pérez, Emilio García-Cabrera, Carlos Jiménez-Juan, Susana Sánchez-Fidalgo

**Affiliations:** 1 Unidad de Gestión Clínica de Farmacia, Hospital Universitario Virgen del Rocío, 41013 Sevilla, Spain; 2 Departamento de Enfermedades Infecciosas, Microbiología y Parasitología, Grupo de Investigación en Enfermedades Infecciosas, Instituto de Biomedicina de Sevilla, Universidad de Sevilla/Consejo Superior de Investigaciones Científicas/Hospital Universitario Virgen del Rocío, 41013 Sevilla, Spain; 3 Centro de Investigación Biomédica en Red de Enfermedades Infecciosas, 28029 Madrid, Spain; 4 Departamento de Medicina Preventiva y Salud Pública, Universidad de Sevilla, 41009 Sevilla, Spain; 5 Unidad de Gestión Clínica Medicina Interna, Hospital Universitario Virgen del Rocío, 41013 Sevilla, Spain

**Keywords:** deprescription, questionnaire, clinicians, barriers

## Abstract

Background and objective: There are barriers to deprescription that hinder its implementation in clinical practice. The objective of this study was to analyse the main barriers and limitations of the deprescription process perceived by physicians who care for multipathological patients. Materials and methods: The “*deprescription questionnaire of elderly patients*” was adapted to an online format and sent to physicians in geriatrics. Question 1 is a reference to establish agreement or disagreement with this practice. The influence of different aspects of deprescription was analysed via the demographic characteristics of the clinicians and perceptions of the various barriers (questions 2–9) by means of bivariate analysis. Based on the latter, a multivariate model was carried out to demonstrate the relationship between barriers and the degree of deprescription agreement among respondents. Results: Of the 72 respondents, 72.2% were in favour of deprescribing. Regarding the analyses, the demographic characteristics did not influence rankings. The deprescription of preventive drugs and consensus with patients were associated with a positive attitude towards deprescribing, while withdrawing drugs prescribed by other professionals, time constraints and patient reluctance emerged as possible barriers. The only factor independently associated with deprescribing was lack of time. Conclusions: Time was found to be the main barrier to deprescription. Training, the creation of multidisciplinary teams and integrated health systems are key facilitators.

## 1. Introduction

Population aging, in recent years, has led to the appearance of a greater number of patients with chronic diseases. These patients are characterised by a high degree of polypharmacy, which is related to a high therapeutic burden and an increased risk of potentially inappropriate medication prescriptions (PIMs) [1]. To combat these PIMs, different strategies can be implemented, among which deprescription stands out.

Deprescription is the review and evaluation of the long-term therapeutic plan, which allows suspending, substituting or modifying the dosage of drugs that were prescribed appropriately but which, under certain clinical conditions, may be considered unnecessary or with an unfavourable benefit–risk ratio [2].

This practice, increasingly established in clinical practice, should be a patient-centred approach that allows patients to participate in making decisions about their pharmacological treatment [3,4].

Despite being an increasingly widespread activity with evidence of its benefits, deprescription has limitations that make it difficult to implement in clinical practice. It is necessary to understand these limitations to carry out the process based on them and to improve the process [5,6].

Many authors are focused on the identification and analysis of the barriers and the facilitators to achieving a more effective deprescription [7,8]. Recently, our group back-translated a structured questionnaire on deprescription into Spanish called the “*Deprescription Questionnaire in the Elderly Patient*” [9]. It was created by a group of Italian researchers based on a bibliographic search for the main barriers to deprescription and allows, through an analysis of the degree of agreement with certain statements, the detection of the main drawbacks found in the implementation of the process.

The objective of this study was to analyse the main barriers and limitations of the deprescription process perceived by physicians who care for these multipathological patients. In this way, critical points that may affect the deprescription process were identified.

## 2. Materials and Methods

The online version of the back-translated questionnaire [9] was carried out using the Google Form application (forms.google.com). It kept the answer options format of the original version, that is, a 7-point Likert scale, in which 1 meant completely disagree and 7 meant completely agree.

The questionnaire remained available for a month on an online server that ensured the confidentiality and anonymity of all respondents. An invitation to participate was sent via email to clinical professionals in various centres that cared for elderly patients. The email also asked the recipients to share the survey with other professionals who might be interested in participating.

The following demographic variables were collected: sex, age, clinical specialty and type of workplace.

The questions were designed to identify the possible barriers to deprescription by prompting respondents to express the specific difficulty of performing deprescription in various scenarios (Supplementary Material Table S1).

Question 1 was established to determine the overall attitude of the respondent towards the process. Respondents who scored between 1 and 5 on the question would be considered “against deprescription or with less confidence to carry it out”, and those who scored 6 or 7 would be considered “in favour of deprescription”. Similarly, the rest of the questions were dichotomized from 1 to 5 and were considered “disagree”, and answers from 6 to 7 were considered “agree”.

### 2.1. Statistical Analysis

The description of the score of the nine questions was expressed using the median and the interquartile range (IQR), as well as the absolute frequencies and percentages of the dichotomous score. A bivariate analysis of the first dichotomized question (“against deprescription/in favour of deprescription”) was performed with the demographic characteristics and the remaining eight questions. We also evaluated whether the initial question was associated with any of the other questions in the questionnaire. For this purpose, we conducted a bivariate analysis of the questions. To assess the association, the chi-square test or Fisher’s test was applied when appropriate. To independently evaluate the association of the barriers identified with the opinion “in favour” and “against” deprescription, a binary logistic regression was performed. In logistic regression, the independent variable introduced was the first dichotomized question, and the introduced covariates were the questions that showed significance in the bivariate analysis. All variables with an association less than or equal to 0.1 were included in the model in the bivariate analysis. The model was performed using a backward step method, adjusted by the Wald statistic, with entry and exit criteria of 0.1 and 0.05, respectively. All statistical tests were performed with the R software, version 4.1.2.

### 2.2. Ethics Approval

The study was conducted in accordance with the Declaration of Helsinki and approved by the Ethics Committee of Virgen del Rocío-Macarena (protocol code FIS-CAL-2019-01 date of approval 2020-03-05).

## 3. Results

### 3.1. Characteristics of the Respondents

A total of 72 physicians answered the online deprescription questionnaire regarding elderly patients, of whom 48 were women (66.7%) and the remaining 24 were men (33.3%). The median age was 59 (IQR: 42–62) years. Seventy-five percent of the participants (*n* = 54) were family doctors, 18.1% (*n* = 13) were internists and 6.9% had other specialties (*n* = 5).

### 3.2. Answers to the Questionnaire

The median and IQR of the responses obtained for each question in the questionnaire are shown in Table 1.

The proportion of specialists based on the response to each question is shown in visual graphic format in Figure S1 (Supplementary Material).

A total of 72.2% of physicians stated that they felt very prepared to perform deprescription in the elderly population (question 1).

Specifically, in patients with limited life expectancy, 83.3% of doctors are in favour of deprescribing drugs with preventive action when the benefits are not justified (question 2). This figure was lower when it came to drugs with therapeutic action (59.7%) (question 3). A total of 48.6% of doctors seemed reluctant to withdraw medications prescribed by other professionals (question 5), 50% were reluctant to deprescribe medications when the patient considered them necessary (question 7), and 68% affirmed that they had no difficulty in agreeing with the patient/caregiver on deprescription strategies (question 9).

It should be noted that three of the questions were formulated in reverse. In this way, the response graphs for questions 4, 6 and 8 show higher response percentages for lower scores (Supplementary Material Figure S1). Thus, it is interesting to compare the scores of the extremes in these cases (responses of 6–7 vs. 1–2). We observed that 33.3% found it difficult to deprescribe preventive drugs when there was no solid evidence to guide how to proceed (question 4), compared to 25% who did not agree with the statement. Similarly, 11.1% of those surveyed stated that they did not deprescribe for fear of the possible adverse effects associated with withdrawal (question 8), while 36.1% believed the opposite. Regarding question 6, the lack of time to carry out this activity, 26.4% of the respondents said they did not have enough time compared to 27.8%, who indicated they had the necessary time.

### 3.3. Analysis of the Responses

The results of Table 2 show that there are no differences between the demographic characteristics of the prescribers who participated in the survey and the fact that they are more in favour of carrying out deprescription.

### 3.4. Qualitative Variables Are Expressed as Numbers (%); Median Quantitative (IQR)

The results of the bivariate analysis of the degree of agreement on deprescription and the possible barriers raised in the remaining questions are shown in Table 3.

When evaluating the association of the degree of trust in deprescribing, we see that in five of the questions, there is an association between the perception of possible barriers and the degree of trust in the deprescription process in general. This occurs, in particular, regarding the barriers raised in questions 2, 5, 6, 7 and 9, where agreement with these questions and the situations that characterise them differs significantly between those who are in favour and against deprescription in general (Table 3).

Thus, when considering whether they would deprescribe drugs with therapeutic action for patients with limited life expectancy, 45% of the respondents from the group least confident in the deprescribing process were in favour, even if continuing the medication was recommended by the guidelines (question 3). Similarly, although the majority of respondents agreed with deprescription in these cases (65%), 35% of them were reluctant.

The results of question 4 show that half of the respondents who were less favourable towards deprescription indicated that it is difficult for them to deprescribe preventive drugs if there is no solid evidence, as did 26.9% of the respondents who were more favourable toward the process.

Regarding the concern about the possible adverse effects associated with withdrawal as a barrier to the process (question 8), 9.6% of the most favourable deprescription group and 15% of the least favourable group agreed with the statement.

In the multivariate analysis, the questions independently associated with deprescription were 2, 6 and 9 (Table 4). Therefore, no independent association was obtained in the degree of agreement for the remaining questions included in the analysis (numbers 4, 5 and 7), referring to the lack of solid evidence, contradicting another specialist or confronting patients and/or caregivers reluctant to change, respectively.

## 4. Discussion

This study evaluated, through a structured questionnaire, the limitations of deprescription in clinical practice and the perception of clinicians regarding this activity to identify possible barriers and/or facilitators.

First, based on the results, the characteristics of the prescriber (age, sex or clinical specialty) do not seem to influence the deprescription process.

This and the other questionnaire responses underscore the important aspects of the process. The first question, which refers generically to the deprescription process, shows a high degree of agreement in favour of this practice among professionals who find it beneficial [10]. Furthermore, these results are in line with the acceptance obtained by clinicians in recent studies, where deprescription has been incorporated into clinical practice [11,12]. Therefore, we can affirm that the trend is positive, that deprescription is gaining support, and that more professionals are advocating its implementation.

Withdrawing drugs in patients with limited life expectancy is the subject of questions 2 and 3. However, number 2 specifies deprescribing preventive drugs, while the third specifies therapeutic drugs. This detail makes a difference in the perception of clinicians. Thus, although the majority of respondents, regardless of whether they are more or less in favour of deprescription, seem to agree on the withdrawal of drugs in this population, only the deprescription of prophylactic drugs is independently associated with the favourable opinion of deprescription. This finding is in line with the literature [13,14].

The question regarding the lack of solid evidence (question 4) obtained some variability in the scores given by the respondents, with 33.3% affirming that this presents a certain conflict when considering deprescription because there is no evidence to support continuing or suspending drugs with preventive purposes. Although the question also deals with prophylactic drugs (like question 2), it differs in the target population, since question 4 does not specifically refer to patients with limited life expectancy. Thus, respondents seem to agree that deprescription is advisable regardless of what is stated in the guidelines or the clinical evidence when life expectancy is limited (question 2). However, when the prognosis of the patients is not mentioned, it seems that the lack of evidence plays a more relevant role. Thus, those who support the deprescription process also have a certain reluctance to carry it out when the evidence is limited and when the patient is not at their end of life. However, the analysis did not conclude that the absence of evidence was decisive for the overall assessment in favour of deprescription.

The fear of withdrawing drugs prescribed by other professionals (question 5), the lack of time (question 6), the reluctance of the patient/caregiver (question 7) and the fear of the adverse effects associated with withdrawal (question 8) are the barriers to the process of deprescription that have been most frequently identified in the literature [5,15,16]. Of these, fear of the adverse effects of withdrawal as a barrier was the only question that did not obtain significant differences between the respondents who were for or against deprescription. It is important to highlight the low number of respondents who, in general, agreed with this statement. Therefore, this fact does not seem to be a limitation. With respect to the rest, deprescribing drugs prescribed by other professionals and the reluctance of patients and caregivers could both be significant barriers to deprescription. Regarding limited time, this aspect seems to be a real barrier to deprescription, as reflected in the literature, affecting the feasibility of its implementation.

Finally, the last question in the questionnaire focuses on the degree of difficulty that physicians have when it comes to reaching a consensus with the patient or their relatives on how to carry out deprescription. This question is more focused on considering the patient as the centre of the healthcare process and considering his or her opinion before making any changes related to their medical care. Although a significant difference was obtained between the respondents who were in favour or against deprescription, due to the large number of clinicians in favour of the process, the multivariate analysis showed that agreeing on a treatment plan with patients and caregivers does not truly imply a problem.

Although it is clear that all barriers, in one way or another, can influence the deprescription process, the appearance of certain barriers in the process affects the feasibility of implementing this activity [15]. The difficulty of modifying prescriptions made by other professionals and the resistance to withdrawal by the patient or caregiver are related to the degree of acceptance of the recommendations by the clinician, a variable highly evaluated in feasibility studies [17,18,19].

As the main limitation of this analysis, the sample size was small and was not obtained randomly. It is likely that the majority of physicians who are more willing to consider deprescription showed greater interest in participating, which could have influenced the results. The establishment of categorization in two unbalanced groups could also be considered a limitation since only scores higher than 5 on the Likert scale were considered positive results. However, this same analysis was performed while considering a value of 4 as “neutral,” and scores of 5 to 7 as “agree”, and the results were practically unaffected. Therefore, we do not believe that it is a real limitation, but rather that it has allowed us to analyse the results based on clearer positions on the part of the clinicians.

On the other hand, the questionnaire chosen was not aimed at identifying barriers in an explicit way, and thus, the interpretation of certain questions formulated in a negative direction made it difficult to understand them. Finally, it should be noted that the barriers to the deprescription process are not limited to what is perceived by physicians, and thus it would be convenient for future studies to complete the analysis with the opinions of patients and other healthcare professionals involved in the process [20].

## 5. Conclusions

By way of conclusion, it could be said that barriers to deprescription exist among physicians; however, they manifest mainly in physicians who are reluctant to deprescribe, while clinicians who consider it a beneficial practice manage the deprescription process. Thus, we consider that the opinion a doctor has regarding this activity is key and greatly influences the appearance of barriers and affects the deprescription process.

It is essential that the doctor is aware of deprescription and supports its implementation. To do this, training, information and evidence are needed. Physicians also need more time to carry out deprescription, including strategies in prescription aid systems and counting. With the support of a pharmacist on the team, these are key aspects to enhance the development and applicability of deprescription.

## Figures and Tables

**Table 1:** Results of the questionnaire.

Deprescription Questionnaire	Median, IQR	Proportion of Respondents with 1–5 Responses (*n*, %)	Proportion of Respondents with 6–7 Responses (*n*, %)
1. Feel confident with deprescribing	6 (5–7)	20 (27.8)	52 (72.2)
2. Preventive deprescription action *	7 (6–7)	12 (16.7)	60 (83.3)
3. Therapeutic deprescription action *	6 (4–7)	29 (40.3)	43 (59.7)
4. No solid deprescription evidence **	4.5 (2.75–6)	48 (66.7)	24 (33.3)
5. Deprescribing other professional prescription	6 (4–7)	35 (48.6)	37 (51.4)
6. Lack of time	4.5 (2–6)	53 (73.6)	19 (26.4)
7. Reluctant patient (need)	5.5 (4.75–6)	36 (50)	36 (50)
8. Fear of adverse effects from withdrawal	3 (2–4)	64 (88.9)	8 (11.1)
9. Difficulty in reaching a consensus with patient/caregiver	6 (5–7)	23 (31.9)	49 (68.1)

* Both questions related to patients with limited life expectancy. ** Specifically, for drugs for preventive purposes.

**Table 2:** Analysis of the demographic characteristics of the respondents and their degree of agreement with the deprescription.

		Feel Confident with Deprescribing Process		
	*n*	Disagree *n* = 20 (%)	Agree *n* = 52 (%)	*p* Value
**Age, median (IQR), *n* = 71**		58.5 (23)	60 (21)	0.513
**Age ranges, *n* = 71**				0.776
25 to 49 years	22	6 (30)	16 (30)	
50 to 59 years	14	5 (25)	9 (16.7)	
60 to 67 years	35	9 (45)	26 (50)	
**Sex, *n* = 72**				0.457
Female	48	12 (60)	36 (69.2)	
Male	24	8 (40)	16 (30.8)	
**Specialty, *n* = 72**				0.167
Family	54	15 (75)	39 (75)	
Internist	13	2 (10)	11 (21.2)	
Other	5	3 (15)	2 (3.8)	

**Table 3:** Bivariate analysis between dichotomized question 1 and dichotomized questions 2 to 9.

		Feel Confident with Deprescribing Process		
Question Number and Barrier	Agree *n*	Disagree *n* = 20 (%)	Agree *n* = 52 (%)	*p* Value
2. Preventive deprescription action *	60	13 (65)	47 (90.4)	0.016
3. Therapeutic deprescription action *	43	9 (45)	34 (65.4)	0.114
4. No solid deprescription evidence **	24	10 (50)	14 (26.9)	0.063
5. Deprescribing other professional prescription	37	5 (25)	32 (61.5)	0.005
6. Lack of time	19	10 (50)	9 (17.3)	0.005
7. Reluctant patient (need)	36	6 (30)	30 (57.6)	0.035
8. Fear of adverse effects from withdrawal	8	3 (15)	5 (9.6)	0.667
9. Difficulty in reaching a consensus with patient/caregiver	49	6 (30)	43 (82.7)	<0.001

* Both questions related to patients with limited life expectancy. ** Specifically, for drugs for preventive purposes.

**Table 4:** Logistic regression model of potential deprescription barriers, unadjusted and adjusted model.

Question No. Barrier	OR	95% CI		
		Lower	Upper	Significance
Unadjusted Model				
2. Preventive deprescription action	0.19	0.03	1.09	0.063
5. Deprescribing other professional prescription	0.45	0.09	2.01	0.305
6. Lack of time	3.91	0.96	15.85	0.057
7. Reluctant patient (need)	0.78	0.17	3.59	0.752
9. Difficulty in reaching a consensus with patient or caregiver	0.11	0.03	0.44	0.002
Adjusted Model				
2. Preventive deprescription action	0.13	0.02	0.65	0.013
6. Lack of time	4.19	1.08	16.45	0.040
9. Difficulty in reaching a consensus with patient or caregiver	0.09	0.02	0.36	0.001

## Data Availability

The data presented in this study are available on request from the corresponding author.

## Supplementary Materials

The following supporting information can be downloaded at: https://www.mdpi.com/article/10.3390/healthcare11131879/s1. Table S1: Description of the barriers raised in each of the questions; Figure S1: Physician responses to the questionnaire.
