# Supplementary material for: The Barriers to Deprescription in Older Patients: A Survey of Spanish Clinicians
Source: Healthcare (Basel). 2023 Jun 29;11(13):1879. doi: 10.3390/healthcare11131879 (PMC10341199; doi:10.3390/healthcare11131879)

Table S1. Description of the barriers raised in each of the questions.

No.	Question	Barrier/opportunity raised
2	I am in favour of deprescribing drugs with preventive action in elderly patients when their life expectancy no longer justifies the possible benefits.	Preventive action drugs in limited life expectancy
3	In the elderly patient with limited life expectancy, it might be appropriate to consider deprescribing some drugs with therapeutic activity, even though these are recommended by clinical practice guidelines.	Medicines of therapeutic action in limited life expectancy
4	In elderly patients, it is difficult for me to consider deprescribing if there is no strong evidence to continue or discontinue drugs for preventive purposes.	Lack of evidence in preventive action drugs
5	I have no problem deprescribing drugs in elderly patients when they were initially prescribed by another health care professional.	Medications prescribed by other professionals
6	Although I consider the deprescription of medications important, I cannot dedicate the necessary time to the patients or their caregiver to do it properly.	Lack of time
7	I have no problem deprescribing medications, and I would suggest it, even if the patients and/or their caregiver thought it would be appropriate to continue taking them.	Patient/caregiver reluctance
8	Although the deprescription of drugs in elderly patients may be considered appropriate in certain situations, I do not do it for fear of possible side effects associated with withdrawal.	Fear of adverse withdrawal effects
9	I have no difficulty in agreeing with patients and/or their caregivers on drug deprescription strategies.	Difficulty in reaching a consensus with the patient/caregiver

Figure S1. Physician responses to the questionnaire.

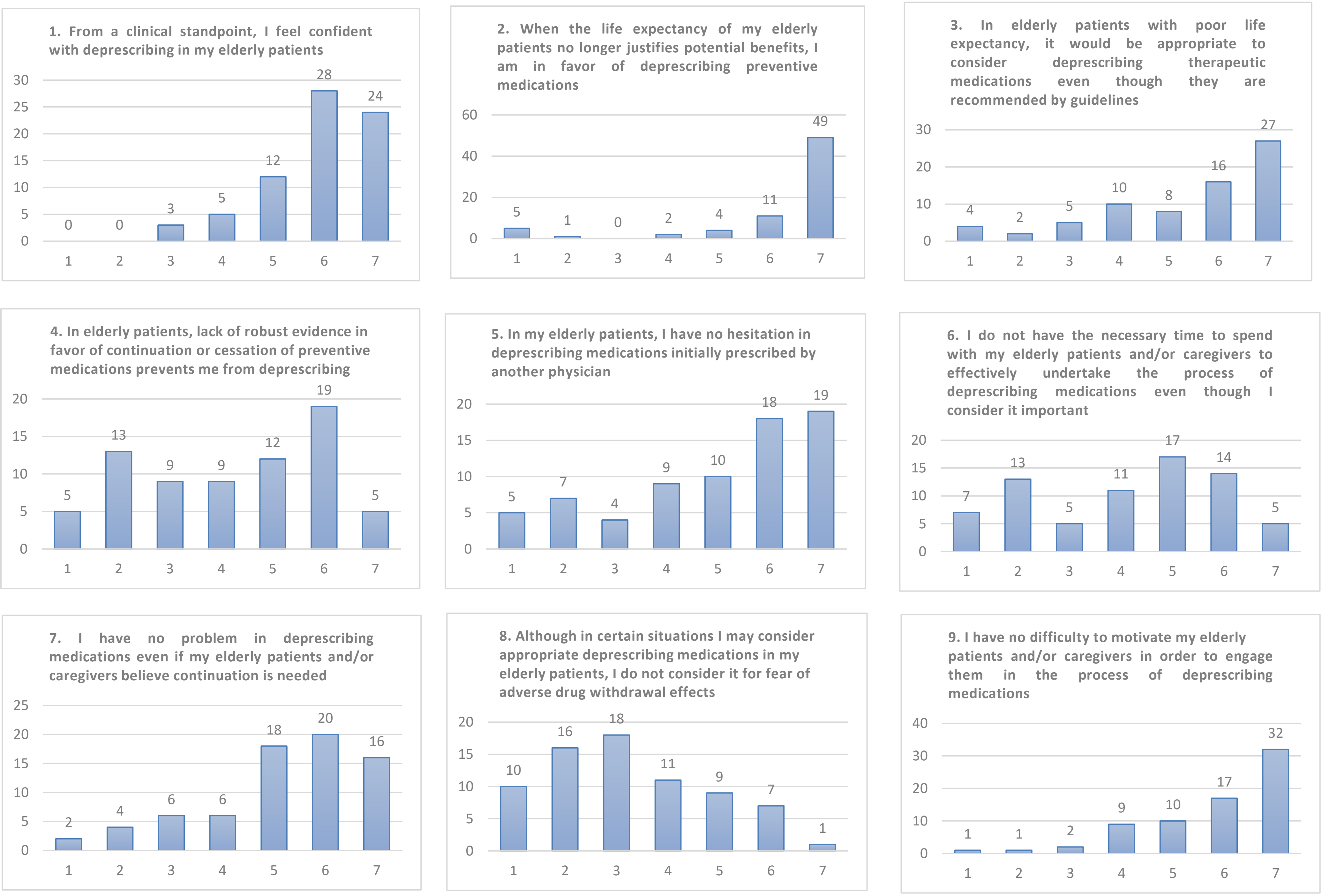

Supplement: Supplementary file 1 [file healthcare-11-01879-s001.zip › healthcare-2321161-supplementary.pdf]
